# Oral health status and socio-behavioral predictors among the Lodha tribe in Odisha, India: a community-based cross-sectional study

**DOI:** 10.3389/fpubh.2026.1889658

**Published:** 2026-10-26

**Authors:** Sujit Ranjan Sahoo, Anuroopa Mohapatra, Abinash Pattanaik, Himadri Gourav Behuria, Sashibhusan Dash, Prativa Panda

**Affiliations:** 1 Department of Dentistry, PRM Medical College and Hospital, Baripada, Odisha, India; 2 Multidisciplinary Research Unit, PRM Medical College and Hospital, Baripada, Odisha, India; 3 PRM Medical College and Hospital, Baripada, Odisha, India

**Keywords:** health equity, indigenous population, oral health, oral leukoplakia, periodontal diseases, vulnerable populations

## Abstract

**Background:**

Socio-historical marginalization drives health disparities among the Lodha tribe, a Particularly Vulnerable Tribal Group (PVTG) in Odisha. The lack of baseline oral health epidemiological data limits targeted health planning. This study aimed to determine oral disease prevalence, oral hygiene status, and socio-behavioral determinants of treatment urgency among the Lodha PVTG in Mayurbhanj, Odisha.

**Methods:**

A community-based cross-sectional survey (*N* = 346 adults) was conducted across four administrative blocks in the Mayurbhanj District using a multi-stage cluster sampling design. Data were collected using modified WHO questionnaires and Type III clinical examinations. Clinical metrics included the Decayed, Missing, and Filled Teeth (DMFT) index, Simplified Oral Hygiene Index (OHI-S), and Community Periodontal Index (CPI). Multivariate binary logistic regression used to identify independent predictors of prompt/immediate treatment urgency.

**Results:**

Participants had a mean OHI-S score of 3.42 ± 1.12, with 61.3% presenting poor oral hygiene. Untreated decay almost entirely drove the mean DMFT of 0.68 ± 1.42 (DT = 0.61 ± 1.31; FT = 0.003 ± 0.05). Periodontal disease was observed in 62.7% (*n* = 217) of participants, with 40.5% demonstrating moderate (4–5 mm) pockets and 22.2% demonstrating severe (≥6 mm) pockets. Oral mucosal lesions affected 33.5% of the sample, with oral leukoplakia dominating cases (9.0%). Dental service utilization remained low, as 89.6% had never visited a dentist. Logistic regression identified tobacco use (aOR = 3.42, 95% CI: 2.11–5.54, *p* < 0.001), poor self-perceived health (aOR = 2.15, 95% CI: 1.28–3.61, *p* = 0.004), and age ≥65 years (aOR = 1.87, 95% CI: 1.05–3.33, *p* = 0.03) as independent predictors of treatment urgency.

**Conclusion:**

The Lodha PVTG exhibits a high burden of untreated periodontal disease and oral precancerous lesions alongside minimal dental service utilization. Targeted oral healthcare services and tobacco cessation programs should be included in tribal development micro-projects to address these disparities.

## Introduction

The morbidity and healthcare exclusion rates of indigenous populations in India are disproportionately high (1, 2). The Government of India recognizes 75 Particularly Vulnerable Tribal Groups (PVTGs) among the Scheduled Tribes that constitute 8.6% of the national population based on pre-agricultural technology, stagnant or declining population, extremely low literacy, and subsistence economy (3, 4).

Odisha has the highest number (total 13) of PVTGs in the Indian states. The Lodha tribe of Mayurbhanj district is socio-economically marginalized, has high illiteracy, and depends on minor forest produce within the Similipal Biosphere Reserve (4–6).

Studies reveal that the tribal groups (PVTG populations) in India have a considerable burden of oral diseases (7). Despite oral health being a significant biomarker of systemic health, with direct correlation of chronic periodontal inflammation with cardiovascular disease, glycemic dysregulation and adverse pregnancy outcomes, it is grossly neglected in tribal primary care, making the populations vulnerable to serious complications (8, 9). The overall oral health profile of Indian tribal populations reveals a severe, multifactorial disease burden, with high rates of dental caries, widespread periodontal destruction, and notable prevalence of Oral Potentially Malignant Disorders (OPMD) (10–15). Various tribal communities of Odisha and surrounding states, including Juang, Kutia Kandha, Birhor, and Irula groups, have traditional oral hygiene practices, such as the use of botanical twigs (e.g., Neem, Sal, or Khajuri) and abrasive agents. Concurrently, the extensive daily consumption of smokeless tobacco products (such as khaini, gutkha, paan, and gudakhu) and indigenously produced alcohol (such as Handia, Mahua, or Salap) creates high-risk environments for oral mucosal pathologies, advanced periodontal destruction, and potential malignant transformation (2, 16, 17).

Despite these recognized risk factors, baseline oral health data for the Lodha PVTG are entirely lacking. Routine national health surveys (such as NFHS-5) do not incorporate detailed clinical dental examinations, leaving a critical evidence gap regarding caries, periodontal status, and mucosal lesion prevalence in this population (18). Designing objective, community-specific oral health interventions requires empirical baseline data. Therefore, this study aimed to determine the prevalence of oral diseases, quantify hygiene indices, and identify socio-behavioral determinants of treatment urgency among the Lodha PVTG of Mayurbhanj, Odisha.

## Materials and methods

### Study design and setting

This community-based cross-sectional epidemiological survey conducted between May 3, 2024, and November 2, 2025, among the Lodha PVTG residing in forest hamlets of Mayurbhanj district, Odisha, India.

### Multi-stage sampling procedure and participant recruitment

A multi-stage cluster sampling design guided participant recruitment across four administrative stages:

1. Stage 1 (Block Selection): The research team purposively selected four administrative blocks—Suliapada, Morada, Udala and Kaptipada based on official Lodha population density records from the Lodha Development Agency (LDA).
2. Stage 2 (Gram Panchayat & Hamlet Selection): Within these four blocks, simple random sampling selected 6 Gram Panchayats (GPs). From these GPs, Probability Proportional to Size (PPS) sampling selected 12 isolated forest hamlets (clusters) (Figure 1).
3. Stage 3 (Participant Recruitment): Field staff conducted systematic door-to-door sampling within each selected hamlet and invited all eligible adult household members (≥18 years) present during survey visits to participate.
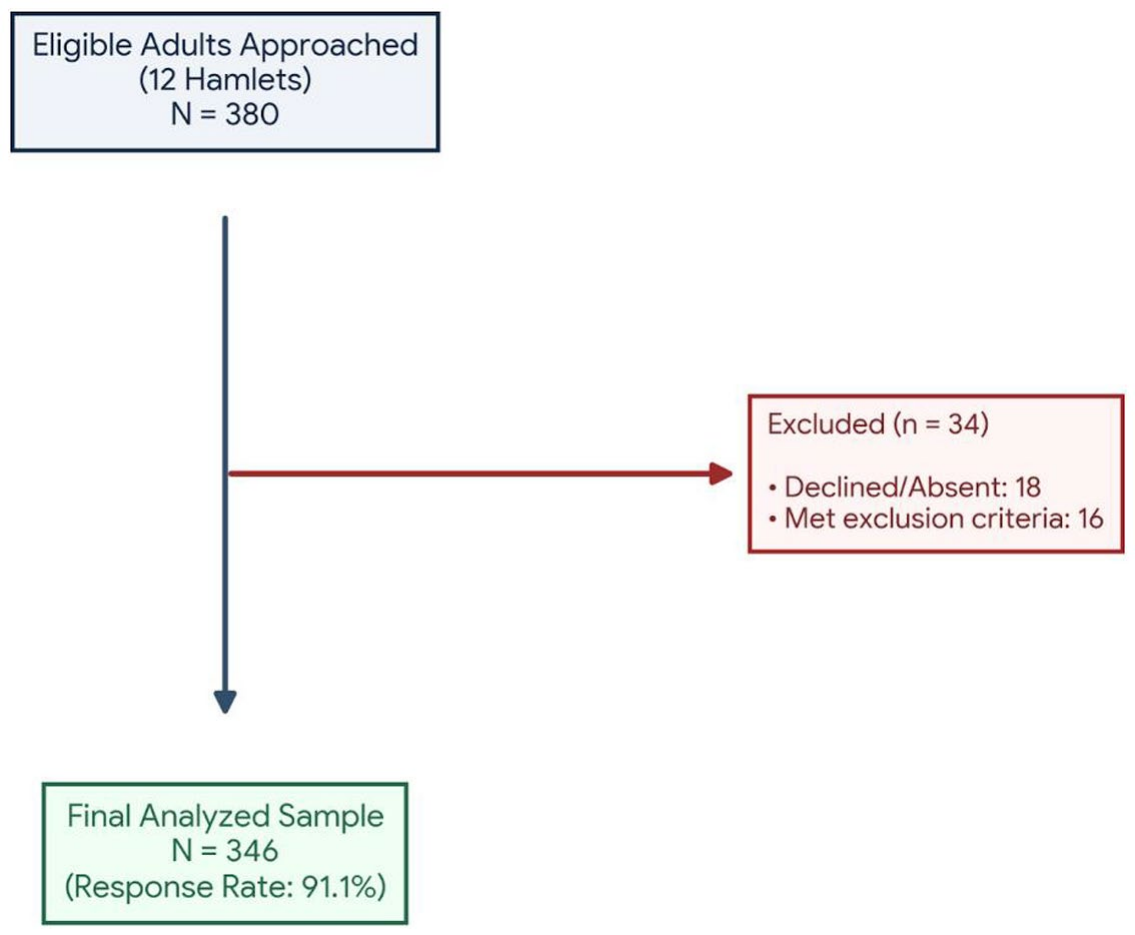

**Figure 1:**
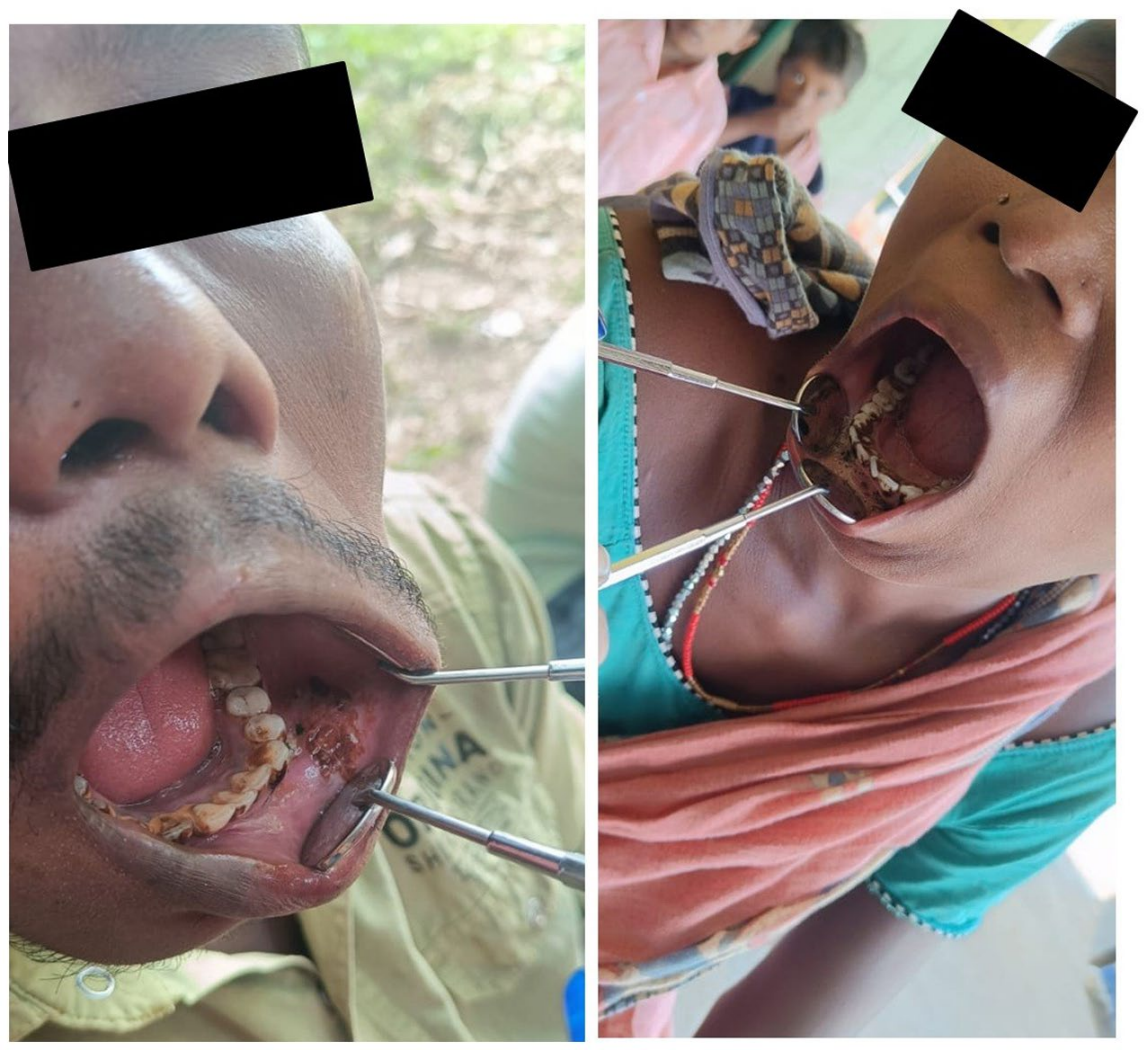
Fieldwork operations and participant recruitment in forest hamlets: Research team conducting door-to-door sampling and household screening within an isolated Lodha tribal settlement in the Mayurbhanj district, Odisha.

### Sample size determination

Cochran’s single proportion formula determined the sample size:

$$n=Z^{2}.P(1-P)\;d^{2}$$

Assumptions: Regional tribal literature established the anticipated prevalence (P) of common oral conditions at 70%, with a 95% confidence level (Z = 1.96), and absolute precision (d) set at 5% (0.05) (16).

Base Sample Size: *n* = 1.96^2^ × 0.70 × 0.30/0.05^2^ = 322.7 approx 323.

Design Effect Adjustment: A design effect (D) of 1.15 adjusted for cluster sampling variability across hamlets, yielding n’ = 323 × 1.15 = 371.45. Accounting for an anticipated non-response/inaccessibility rate of ~10% established a target sample size of 410. Field operations and geographical constraints produced a final enrolled sample of *N* = 346, which preserved statistical power (>80%) for primary prevalence estimates.

### Inclusion and exclusion criteria

- Inclusion Criteria: Self-identified Lodha PVTG adults (≥18 years), continuous residence in the study area for ≥6 months, and provision of written/thumbprint informed consent.
- Exclusion Criteria: Individuals requiring antibiotic prophylaxis prior to periodontal probing, severe cognitive or physical limitations preventing clinical assessment, acute systemic medical emergencies, and currently pregnant women (to prevent misclassifying pregnancy-induced gingival enlargement and transient pseudo-pockets as true attachment loss/periodontitis).

### Examiner calibration and reliability

Two trained, calibrated dental examiners—assisted by local ASHA workers and community facilitators conducted all clinical examinations. Prior to field data collection, training and calibration exercises evaluated a sample of 20 non-study subjects displaying varied oral pathologies. Calibration yielded intra-examiner reliability Kappa (κ) coefficients of 0.86 and 0.88 for DMFT and CPI scoring, respectively. Inter-examiner reliability produced a κ coefficient of 0.84 for CPI scoring and 0.85 for mucosal lesion identification, demonstrating high diagnostic consistency.

### Data collection and operational definitions of clinical parameters

Data collection relied on a structured, face-to-face questionnaire adapted from the WHO Oral Health Survey for Adults (2013), translated and back-translated into Odia. Examiners performed clinical assessments under natural daylight supplemented by portable LED headlamps (Type III examination) using WHO CPI probes and plane mouth mirrors. Standard infection control procedures (glutaraldehyde chemical sterilization and batch autoclaving) governed all equipment handling.

- Simplified Oral Hygiene Index (OHI-S): Combined Debris Index (DI-S) and Calculus Index (CI-S) scores (range 0.0–6.0). Good (0.0–1.2), Fair (1.3–3.0) and Poor (3.1–6.0) oral hygiene status was evaluated using standard cutoff criteria (19).
- Dental Caries (DMFT): The total caries experience was assessed as the sum of Decayed (DT), Missing due to caries (MT), and Filled (FT) permanent teeth (20).
- Gingivitis: Was defined as gingival bleeding on probing (CPI Score 1) or the presence of calculus (CPI Score 2) in the absence of pathological periodontal pockets or attachment loss (20).
- Periodontitis: Was assessed by the Community Periodontal Index (CPI). Pathological pockets ≥4 mm defined periodontitis (CPI Score 3: moderate periodontitis, 4-5 mm; CPI Score 4: severe periodontitis, ≥6 mm) (20).
  - Methodological Justification & Limitations: International standardization and feasibility in resource-limited field settings justified selecting CPI. However, CPI relies on index teeth and may over- or underestimates full-mouth attachment loss compared to comprehensive full-mouth clinical attachment level (CAL) examinations.
- Treatment Urgency: Four standard WHO operational tiers categorized treatment urgency
  - Stage 1: No treatment needed.
  - Stage 2: Routine / preventive care.
  - Stage 3: Prompt treatment (caries involving dentin, severe gingivitis).
  - Stage 4: Immediate / urgent care (acute pain, abscesses and suspicious mucosal lesions).

### Statistical analysis

Data were analyzed using SPSS 27.0 (IBM Corp., Armonk, NY). Mean ± SD summarizes continuous variables (including DMFT and OHI-S), while counts (n) and percentages (%) report categorical data.

Pearson’s Chi-square (χ^2^) test evaluated cross-tabulations—including gender-disaggregated risk behaviors—when expected cell counts equaled or exceeded 5; Fisher’s exact test (including Fisher–Freeman–Halton for RxC tables) analyzed tables with expected counts below 5. Analysis required consolidating low-frequency mucosal categories (Leukoplakia, Other Pathologies, Absent) prior to testing.

Multivariate binary logistic regression identified independent predictors of prompt/immediate treatment urgency (Stages 3–4 vs. Stages 1–2). Biological plausibility and bivariate significance (*p* < 0.10) dictated candidate selection into the model. Variance inflation factors indicated no substantial multicollinearity (VIF < 1.4). The model demonstrated good fit (Hosmer–Lemeshow: χ2 = 5.42, df = 8, *p* = 0.71) and explained 18–25% of variance (Cox & Snell R^2^ = 0.18, Nagelkerke R^2^ = 0.25). Analysis reports adjusted odds ratios (aOR) and 95% confidence intervals.

Two-tailed *p*-values display to two decimal places, while values reaching high statistical significance render as *p* < 0.001.

## Results

### Socio-demographic profile and behavioral risk factors

The analyzed cohort comprised 346 adults (mean age 44.2 ± 14.6 years), with females forming a slight majority. Laborers and homemakers formed the core occupational groups, and nearly two-thirds of the population lacked formal schooling (Table 1).

**Table 1:** Socio-demographic profile and lifestyle risk factors (*N* = 346).

Characteristic	Category	Frequency (n)	Percentage (%)
Sex	Male	153	44.2
	Female	193	55.8
Age group (Years)	18–34	109	31.5
	35–44	63	18.2
	45–64	121	35
	≥65	53	15.3
Occupation	Laborer	107	30.9
	Homemaker	100	28.9
	Forest Leaf Gatherer	60	17.3
	Agriculture / Husbandry	53	15.3
	Student / Government Employee	26	7.5
Education level	No formal schooling	219	63.3
	Primary / Secondary Schooling	109	31.5
	College / University	18	5.2
Tobacco use pattern	Smokeless Tobacco	241	69.7
	Smoking (Bidi / Cigarette)	6	1.7
	Dual User (Smokeless + Smoking)	11	3.2
	Non-User	88	25.4
Alcohol consumption	Daily / Frequent (Handia / Local brew)	242	69.9
	Non-Consumer	104	30.1

The study population used substances widely. Smokeless tobacco (primarily khaini, gutkha, and raw leaf with lime) was the predominant form of tobacco consumption, significantly outstripping exclusive smoking and dual use. Men demonstrated a significantly higher prevalence of smokeless tobacco use compared to women (χ^2^ = 6.21, *p* = 0.01). A clear majority of participants also reported daily consumption of local alcoholic beverages (handia or mahua).

### Oral hygiene practices and dentifrice selection

Traditional practices almost entirely dominated oral hygiene. The vast majority of participants utilized botanical twigs (such as sal, neem, or acacia) as their primary cleaning implement, whereas modern toothbrush adoption remained minimal (Table 2).

**Table 2:** Oral hygiene behaviors and dental care utilization patterns (*N* = 346).

Variable	Category	Frequency (n)	Percentage (%)
Primary hygiene tool	Botanical Twig (Sal / Neem / Acacia)	301	87
	Toothbrush	44	12.7
	None / Finger	1	0.3
Primary dentifrice	Plain Water / Twig Alone	236	68.2
	Toothpaste / Toothpowder	56	16.2
	Tobacco Leaf Preparation (Gudakhu / Local mixture)	44	12.7
	Charcoal / Wood Ash / Mud	10	2.9
Cleaning frequency	Once Daily	336	97.1
	Twice Daily or More	10	2.9
Dental care history	Never Received Professional Care	310	89.6
	Care Received within Past 2 Years	21	6.1
	Care Received >2 Years Ago	15	4.3

Non-standard agents similarly dominated dentifrice selection, with most individuals relying on plain water, natural abrasives (charcoal, wood ash, or mud), or tobacco leaf preparations (gudakhu). Nearly all subjects maintained a single daily cleaning routine. Participants used dental services exceptionally rarely; the vast majority had never visited a dentist, and acute pain served as the primary catalyst among the few who had received prior care.

### Clinical findings, diagnostic indices, and disease burden

Clinical evaluations revealed substantial oral disease burdens across all measured indices (Table 3):

- Oral Hygiene Index-Simplified (OHI-S): The mean cohort score fell into the poor range (3.42 ± 1.12). Over three-fifths of participants exhibited poor debris and calculus accumulation.
- Dental Caries (DMFT): While overall caries experience was relatively low (mean DMFT 0.68 ± 1.42), treatment met virtually no needs; untreated active decay (DT) accounted for 89.7% of the overall index, and 99.7% of individuals with caries had untreated lesions (Figure 2).
- Periodontal Status & Mucosal Lesions: Pathological periodontal pockets (>4 mm) affected nearly two-thirds of the cohort. Clinicians detected oral mucosal pathologies in one-third of participants, driven predominantly by oral leukoplakia, followed by dental abscesses, lichen planus, and suspected oral malignancy.
- WHO Treatment Urgency: More than half of the study population required prompt or immediate clinical intervention (Stages 3 & 4).

**Table 3:** Clinical findings, diagnostic indices, and WHO treatment urgency tiers (*N* = 346).

Metric/parameter	Sub-category/value	Frequency (n)	Percentage (%)
OHI-S categories	Good (0.0–1.2)	28	8.1
	Fair (1.3–3.0)	106	30.6
	Poor (3.1–6.0)	212	61.3
	*Mean OHI-S Score ±SD*	*3.42 ± 1.12*	*—*
Natural teeth presence	>20 teeth	326	94.2
	10–19 teeth	14	4
	1–9 teeth	6	1.7
Periodontal status (CPI)	Healthy (Score 0)	129	37.3
	Moderate Periodontitis (4-5 mm, Score 3)	140	40.5
	Severe Periodontitis (≥ 6 mm, Score 4)	77	22.2
Gingivitis	(Scores 1 & 2)	143	41.3
DMFT index components	Mean Decayed Teeth (DT ± SD)	0.61 ± 1.31	—
	Mean Missing Teeth (MT ± SD)	0.07 ± 0.28	—
	Mean Filled Teeth (FT ± SD)	0.003 ± 0.05	—
	*Total Mean DMFT Index ±SD*	*0.68 ± 1.42*	*—*
Mucosal pathologies	Malignant Tumor	1	0.3
	Oral Leukoplakia	31	9.0
	Lichen Planus	5	1.4
	Acute Dental Abscess	6	1.7
	Suspected Malignancy	1	0.3
	Other condition	72	20.8
WHO treatment urgency	Stage 1: No Treatment Needed	68	19.7
	Stage 2: Routine / Preventive Care	75	21.7
	Stage 3 & 4: Prompt or Immediate Care	203	58.70%

OHI-S, Simplified Oral Hygiene Index; CPI, Community Periodontal Index; DMFT, Decayed, Missing, Filled Teeth; SD, Standard Deviation.

**Figure 2:**
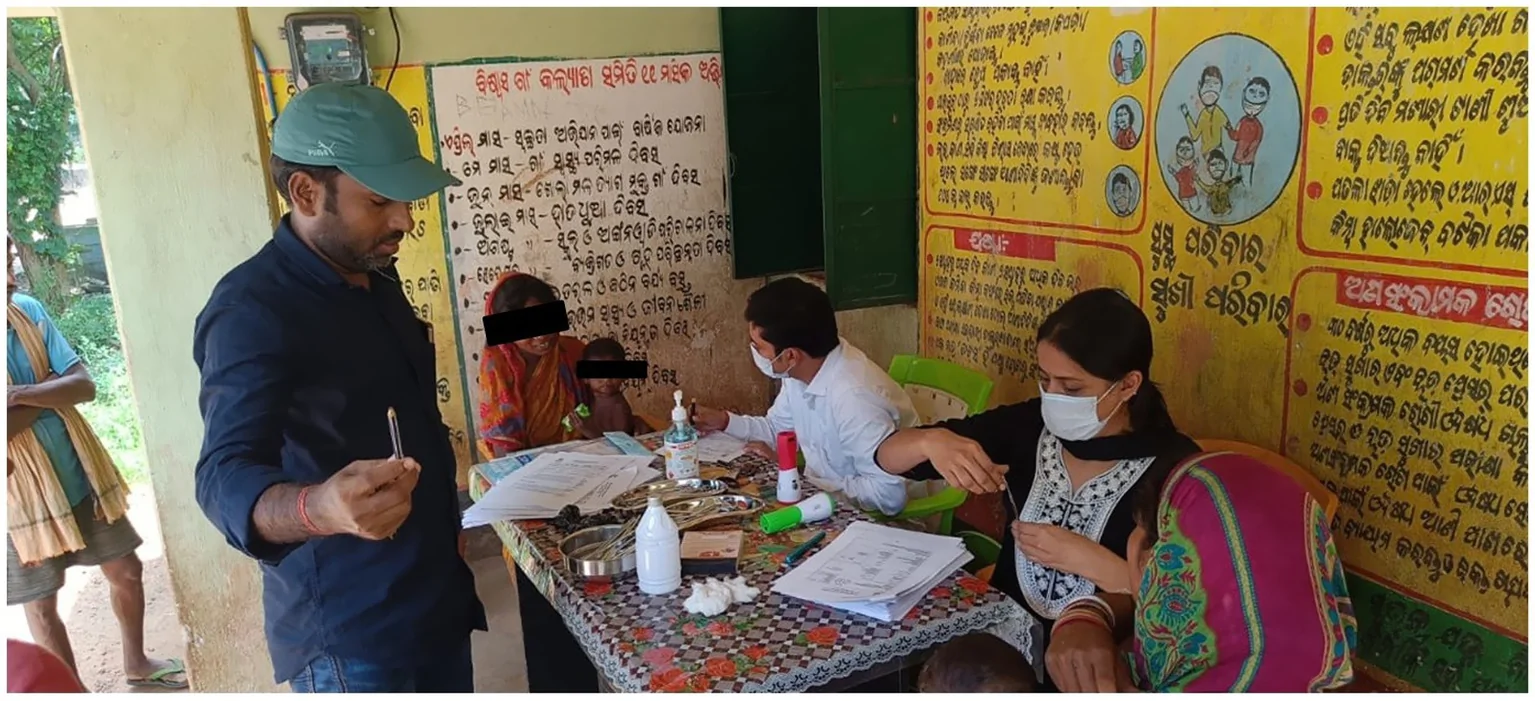
Clinical presentation of oral mucosal lesion in Lodha adults. The intra-oral views demonstrate severe clinical manifestations associated with chronic smokeless tobacco and local alcohol consumption.

### Risk factors and determinants of oral disease

Bivariate analysis identified key sociodemographic and behavioral correlates of disease burden (Table 4). Age and tobacco use associated significantly with both periodontitis (*p* < 0.001) and oral mucosal lesions (*p* < 0.001). There was a significant association between lower educational attainment and higher rates of periodontitis (*p* = 0.02), and a borderline association between alcohol use and mucosal pathologies (*p* = 0.05).

**Table 4:** Bivariate associations of determinants with periodontitis and mucosal lesions (*N* = 346).

Risk factor	Sub-category (n)	Periodontitis present (n)	Periodontitis absent (n)	*p*-value*	Mucosal lesions present †(n)	Mucosal lesions absent (n)	*p*-value*
Sex	Male (153)	99	54	0.14	54	99	0.08
	Female (193)	118	75		62	131	
Age group	18–34 (109)	58	51	**<0.001**	46	63	**<0.001**
	35–44 (63)	35	28		22	41	
	45–64 (121)	82	39		41	80	
	≥65 (53)	42	11		7	46	
Education	Unschooled (219)	155	64	**0.02**	70	149	0.44
	Primary/Secondary (109)	61	48		41	68	
	College/Higher (18)	1	17		5	13	
Tobacco use	Smokeless (241)	166	75	**<0.001**	103	138	**<0.001**
	Smoking/Dual (17)	11	6		4	13	
	Non-User (88)	40	48		9	79	
Alcohol use	Consumer (242)	159	83	0.18	89	153	0.05
	Non-Consumer (104)	58	46		27	77	

*p-values calculated via Pearson’s Chi-square (χ^2^) test or Fisher–Freeman–Halton exact test where cell counts <5. †Composite mucosal lesion presence (Leukoplakia, Lichen Planus, Abscess, Malignancy).

### Factors influencing treatment urgency

The distribution of WHO Treatment Urgency varied significantly by demographic characteristics, tobacco use, self-reported health and presence of mucosal disease (Table 5). A higher proportion of urgent care needs (Stages 3 & 4) were observed among older age groups (*p* = 0.04), among active tobacco users (*p* < 0.001), among those reporting poor general health (*p* = 0.01) and among those with mucosal diagnoses such as leukoplakia, abscesses or suspected malignancy (*p* = 0.02).

**Table 5:** Distribution of WHO treatment urgency across demographics and pathologies (*N* = 346).

Variable	Sub-category (n)	Stage 1: no care (n)	Stage 2: routine care (n)	Stage 3 & 4: urgent care (n)	*p*-value*
Sex	Male (153)	28	33	92	0.68
	Female (193)	40	42	111	
Age group	18–34 Years (109)	32	20	57	**0.04†**
	35–44 Years (63)	15	14	34	
	45–64 Years (121)	15	25	81	
	≥65 Years (53)	6	16	31	
Tobacco status	Tobacco User (258)	41	51	166	**<0.001**
	Non-User (88)	27	24	37	
Perceived health	Excellent / Good (257)	59	58	140	0.01
	Poor / Very Poor (85)	9	17	59	
	Do not Know (4)	0	0	4	
Mucosal diagnosis	Leukoplakia (*n* = 31)	2	5	24	0.02†
	Lichen Planus (*n* = 5)	1	1	3	
	Suspicious /Malignancy (Malignant Tumor) (*n* = 2)	0	1	1	
	Abscess (*n* = 6)	0	0	6	
	Others (*n* = 72)	7	12	53	

*p-values calculated via Pearson’s Chi-square (χ^2^) test. † *p*-values calculated via Fisher–Freeman–Halton Exact Test due to expected cell counts < 5.

### Multivariate predictors of urgent treatment needs

In the fully adjusted multivariate logistic regression model (Table 6), tobacco use was the strongest independent predictor of urgent or immediate treatment need (aOR = 3.42, 95% CI: 2.11–5.54, *p* < 0.001). Individuals reporting poor or very poor perceived health had more than double the odds of requiring urgent intervention compared to those reporting good health (aOR = 2.15, 95% CI:1.28–3.61, *p* = 0.004). Older age (≥65 years) also retained independent significance (aOR = 1.87, 95%CI: 1.05–3.33, *p* = 0.03). Gender and education level did not significantly influence treatment urgency after controlling for covariates.

**Table 6:** Complete multivariate logistic regression output for prompt/immediate treatment urgency.

Variable	Reference category	Comparison group	Unadjusted OR (95% CI)	Adjusted OR (aOR)*	95% CI for aOR	*p*-value
Tobacco use	Non-Users	Tobacco Users	3.28 (1.98–5.41)	3.42	2.11–5.54	<0.001
Perceived health	Excellent / Good	Poor / Very Poor	2.08 (1.21–3.55)	2.15	1.28–3.61	0.004
Age group	18–34 Years	≥65 Years	1.76 0.98–3.12)	1.87	1.05–3.33	0.03
Education level	Schooled	No Formal Schooling	1.39 (0.84–2.28)	1.45	0.89–2.37	0.13
Sex	Female	Male	1.09 0.70–1.68)	1.12	0.72–1.74	0.61

* Model mutually adjusted for all listed variables. Model Fit Statistics: Hosmer–Lemeshow Test χ^2^ = 5.42, df = 8, *p* = 0.71; Cox & Snell R^2^ = 0.18; Nagelkerke R^2^ = 0.25; Max VIF = 1.34. OR, Odds Ratio; aOR, Adjusted Odds Ratio; CI, Confidence Interval; VIF, Variance Inflation Factor.

## Discussion

This community-based cross-sectional study provides baseline evidence on the oral health status and socio-behavioral determinants of the Lodha, a Particularly Vulnerable Tribal Group (PVTG) in Mayurbhanj, Odisha. The findings demonstrate a high burden of untreated oral pathology notably chronic periodontal disease (62.7%), oral leukoplakia (9.0%), and unrestored dental decay (99.7% of all carious lesions) alongside minimal utilization of professional dental care (89.6% never examined by a dentist). These findings align with national trends observed in diverse tribal communities in Odisha (11, 12, 15, 16, 21), Tamil Nadu (22–26), Jharkhand (27, 28), Kerala (29, 30), Telangana (31, 32), West Bengal (33), and Karnataka (34), where the burden of disease has been shown to cluster in the face of structural barriers to healthcare access.

### Socio-demographic patterns and sex disparities

The study population is characterized by high levels of structural disadvantage, including an illiteracy rate of 63.3% and primary reliance on manual labor. These features are consistent with the broader Indian tribal population, where lower levels of education and economic hardship are linked to higher rates of tobacco adoption (11, 17, 23, 24, 29, 30, 35, 36). Prior literature on indigenous groups often describes male-dominant tobacco use, nicotine dependence, and oral precancer burdens (12, 13, 22, 29, 33, 35, 36), as well as a higher burden of untreated caries and periodontitis among women (21, 23, 24, 26, 30). In contrast, univariate analysis in this Lodha cohort demonstrated no statistically significant sex differences in periodontitis (*p* = 0.14), mucosal lesions (*p* = 0.08), or treatment urgency (*p* = 0.68). High rates of smokeless tobacco consumption among both males (76.5%) and females (64.2%) likely explain this convergence by attenuating typical sex-based differences in oral disease presentation. Because females comprised 55% of the study cohort, and this explicitly excluded pregnant individuals at baseline. This step prevented pregnancy-associated gingival enlargement and hormonal pseudo pockets from confounding true periodontal probing depth measurements.

### Oral hygiene practices and mechanical–chemical factors

The majority of participants relied on traditional oral hygiene methods, primarily botanical twigs such as Sal (87.0%). Adherence to modern toothbrushes and fluoridated toothpaste was low (3.5–7.6%), consistent with patterns observed in isolated tribal cohorts in Odisha and Jharkhand (11, 16, 17, 28) but lower than rates reported in parts of Kerala, West Bengal, and Tamil Nadu (23, 24, 29, 30, 33).

While botanical twigs possess intrinsic phytochemical and antimicrobial properties (27, 28, 34), their capacity to disrupt sub gingival plaque remains lower than that of standard manual toothbrushes. Nevertheless, bivariate analysis showed no significant association between the choice of cleaning tool (twig vs. toothbrush) and periodontitis (*p* = 0.10) or mucosal lesions (*p* = 0.44). This indicates that mechanical cleaning tools alone do not offset the persistent biological impact of heavy smokeless tobacco exposure, infrequent oral hygiene practices (97.1% brushing once daily), and lack of professional preventive care.

### Caries experience, dietary contradictions, and restorative neglect

The observed mean DMFT score in this Lodha sample was low (0.68 ± 1.42), with 71.3% of participants presenting as caries-free. Low overall DMFT scores are also documented among the Birhors (DMFT 1.02–1.05) (27) and Koyas (32). However, attributing low caries experience strictly to traditional unrefined diets presents an interpretive contradiction in this cohort: study did not record specific dietary variables (such as fibrous tubers or millets) in the primary results, whereas frequent consumption of commercial sweetened drinks and snacks were documented.

This low DMFT score despite sugary food exposure may reflect limited cumulative exposure window durations, protective salivary buffering, or under-diagnosis of initial smooth-surface lesions in field settings. Conversely, other regional groups display substantially higher caries burdens—such as the Juangs (83.4% caries prevalence) (15), Kutia Kandhas (DMFT 4.06 ± 3.55) (11), Irulars (DMFT 6.58 ± 3.99) (23), and older adult Paniyas (DMFT 18.47 ± 13.10) (29).

Extreme component disparity characterized the DMFT index in this study: the Decayed component (DT = 0.61 ± 1.31) accounted for almost the entire index, while the Filled component was near zero (FT = 0.003 ± 0.05). Among participants with active decay, 99.7% remained unrestored. Universal restorative neglect remains a consistent finding across tribal dental literature (11, 27, 30, 34). Without restorative services, carious lesions continue to progress unchecked to terminal exfoliation or extraction (25, 26).

### Periodontal breakdown, systemic confounders, and mucosal lesions

Pathological periodontal pockets (>4 mm) involved 62.7% of participants. Kutia Kandhas (75.1% with pockets >4 mm) (11), Paniyas (76.9%) (29) and Bharias (74.7%) (13) cohorts with high prevalence of advanced periodontal disease. Age correlated positively with periodontitis severity (*p* < 0.001), reflecting cumulative tissue breakdown over time. Chemical components of smokeless tobacco contribute directly to periodontal degeneration via local tissue toxicity and vasoconstriction, which impair mucosal perfusion and local immune response (37, 38).

Major non-communicable diseases (NCDs), particularly Type 2 diabetes mellitus and hypertension, act as established bidirectional risk factors for periodontal destruction (8, 9). Because we did not perform clinical diagnostic testing for diabetes and blood pressure during this field survey, undiagnosed NCDs represent an unmeasured systemic confounder that could independently exacerbate periodontal breakdown.

Oral mucosal lesions occurred in 33.5% of participants, with oral leukoplakia presenting in 9.0%. High rates of oral potentially malignant disorders (OPMDs) are documented across regional tribal groups, such as Narikuravars (48.3%) (22) and Odisha tribals (27.1%) (12), heavily driven by smokeless tobacco formulations (khaini, gutkha) and local alcohol preparations (11, 12, 17, 30, 35, 36). The leukoplakia prevalence observed in this study exceeds national general population averages (1.5–3.0%) (39). Concurrent alcohol consumption may enhance mucosal permeability to tobacco nitrosamines, increasing lesion development (8, 15, 16, 25, 40).

### Predictors of treatment urgency and self-perceptions of health

Multivariate logistic regression identified tobacco use (aOR = 3.42, 95% CI: 2.11–5.54, *p* < 0.001), self-perceived poor health (aOR = 2.15, 95% CI: 1.28–3.61, *p* = 0.004), and age ≥65 years (aOR = 1.87, 95% CI: 1.05–3.33, *p* = 0.03) as independent predictors of clinical treatment urgency. This supports literature demonstrating that pain, advanced lesion progression, and older age drive urgent care needs in remote settings (11, 13, 28–30, 39).

A mismatch occurred between subjective perception and objective clinical findings: 74.3% of participants rated their oral health as good or average, despite 62.7% presenting active periodontal disease. Isolated communities widely report this normalization of chronic oral symptoms where dental services are absent, health literacy is limited, and dental anxiety or perceived lack of treatment efficacy is common (12, 13, 25, 26). Nevertheless, self-reported poor health correlated significantly with objective periodontitis (*p* = 0.0004) and treatment urgency, supporting the utility of simple single-item self-evaluations for rapid community risk screening. Reported quit intentions among tobacco users (30.1–31.2%) highlight an opportunity for targeted cessation interventions (35).

### Strengths of the study

1. Baseline PVTG Data: Provides clinical epidemiological data on oral disease burden and treatment urgency specific to the Lodha PVTG in Odisha.
2. Sampling Methodology: Employs a multi-stage cluster sampling design across 12 hamlets with a high response rate (91.1%), supporting population-level estimations.
3. Calibrated Assessments: Trained examiners conducted field examinations and demonstrated high inter- and intra-examiner reliability (κ ≥ 0.84).
4. Standardized Protocols: Applied WHO Basic Oral Health Survey protocols to enable direct comparison with regional and national datasets.

### Limitations of the study

1. Cross-Sectional Design: The observational framework precludes establishing causal relationships between behavioral risks and disease development.
2. Periodontal Index Metrics: Use of the WHO Community Periodontal Index (CPI) on index teeth may under- or overestimate full-mouth Clinical Attachment Loss (CAL) relative to comprehensive full-mouth examinations.
3. Unmeasured Systemic Confounders: Researchers did not record or adjust for objective clinical data regarding Type 2 diabetes and hypertension in multivariate models, introducing potential confounding for periodontal outcomes.
4. Self-Report Bias: Behavioral data regarding substance use, oral hygiene tools, and dietary patterns remain subject to recall and social desirability biases.
5. Generalizability: The external validity of these findings relates directly to the socio-geographic context of the study setting. The sampling methodology across 12 hamlets (91.1% response rate) allows generalization to the adult Lodha PVTG population in Mayurbhanj district who share similar socio-economic conditions and healthcare access barriers. These results may also apply moderately to other forest-dependent PVTGs in Odisha (such as the Birhor, Hill Kharia, Mankdia and Juang) that present comparable socio-demographic profiles. Researchers should exercise caution when extrapolating these estimates to non-PVTG tribal or general rural populations with higher literacy, different dietary exposures, and greater access to healthcare services.

## Conclusion

The Lodha PVTG in Mayurbhanj, Odisha, suffers from a severe burden of chronic periodontal disease and oral leukoplakia with a complete lack of utilization of restorative dental care. Tobacco consumption, self-perceived poor health, and advanced age are important independent predictors of urgent treatment needs. To address these profound oral health disparities, there is a need to integrate mobile dental care, routine screening, and community-based tobacco cessation counseling into primary healthcare networks and micro-projects run by the Lodha Development Agency. Subsequent studies should be large, multi-block longitudinal cohort studies to map disease trajectories and elucidate causal pathways. The development of low-cost mobile Health screening tools, the evaluation of the determinants of healthcare access, qualitative studies to identify the drivers of tobacco addiction and the profiling of plaque microbiology, genetic resistance factors and gingival biotypes are key research frontiers. Today, the only way to reduce this disease burden is through the immediate implementation of standardized, culturally appropriate oral health promotion programs, delivered by local tribal health workers and integrated into public health infrastructure.

## Data Availability

The original contributions presented in the study are included in the article/supplementary material, further inquiries can be directed to the corresponding author.
